# Prediction of disease severity in COPD: a deep learning approach for anomaly-based quantitative assessment of chest CT

**DOI:** 10.1007/s00330-023-10540-3

**Published:** 2023-12-27

**Authors:** Silvia D. Almeida, Tobias Norajitra, Carsten T. Lüth, Tassilo Wald, Vivienn Weru, Marco Nolden, Paul F. Jäger, Oyunbileg von Stackelberg, Claus Peter Heußel, Oliver Weinheimer, Jürgen Biederer, Hans-Ulrich Kauczor, Klaus Maier-Hein

**Affiliations:** 1 Division of Medical Image Computing, German Cancer Research Center (DKFZ), Im Neuenheimer Feld 280, 69120 Heidelberg, Germany; 2 Translational Lung Research Center Heidelberg (TLRC), Member of the German Lung Research Center (DZL), Heidelberg, Germany; 3 Medical Faculty, Heidelberg University, Heidelberg, Germany; 4 National Center for Tumor Diseases (NCT), NCT Heidelberg, a partnership between DKFZ and Heidelberg University Medical Center, Heidelberg, Germany; 5 Interactive Machine Learning Group (IML), German Cancer Research Center (DKFZ), Heidelberg, Germany; 6 Helmholtz Imaging, German Cancer Research Center (DKFZ), Heidelberg, Germany; 7 Division of Biostatistics, German Cancer Research Center (DKFZ), Heidelberg, Germany; 8 Pattern Analysis and Learning Group, Radiation Oncology, Heidelberg University Hospital, Heidelberg, Germany; 9 Department of Diagnostic and Interventional Radiology, Heidelberg University Hospital, Heidelberg, Germany; 10 Diagnostic and Interventional Radiology with Nuclear Medicine, Thoraxklinik at University Hospital, Heidelberg, Germany; 11 Faculty of Medicine, University of Latvia, Raina Bulvaris 19, Riga, LV-1586 Latvia; 12 Faculty of Medicine, Christian-Albrechts-Universität zu Kiel, D-24098 Kiel, Germany

**Keywords:** Chronic obstructive pulmonary disease, Deep learning, Artificial intelligence, Computed tomography

## Abstract

**Objectives:**

To quantify regional manifestations related to COPD as anomalies from a modeled distribution of normal-appearing lung on chest CT using a deep learning (DL) approach, and to assess its potential to predict disease severity.

**Materials and methods:**

Paired inspiratory/expiratory CT and clinical data from COPDGene and COSYCONET cohort studies were included. COPDGene data served as training/validation/test data sets (*N* = 3144/786/1310) and COSYCONET as external test set (*N* = 446). To differentiate low-risk (healthy/minimal disease, [GOLD 0]) from COPD patients (GOLD 1–4), the self-supervised DL model learned semantic information from 50 × 50 × 50 voxel samples from segmented intact lungs. An anomaly detection approach was trained to quantify lung abnormalities related to COPD, as regional deviations. Four supervised DL models were run for comparison. The clinical and radiological predictive power of the proposed anomaly score was assessed using linear mixed effects models (LMM).

**Results:**

The proposed approach achieved an area under the curve of 84.3 ± 0.3 (*p* < 0.001) for COPDGene and 76.3 ± 0.6 (*p* < 0.001) for COSYCONET, outperforming supervised models even when including only inspiratory CT. Anomaly scores significantly improved fitting of LMM for predicting lung function, health status, and quantitative CT features (emphysema/air trapping; *p* < 0.001). Higher anomaly scores were significantly associated with exacerbations for both cohorts (*p* < 0.001) and greater dyspnea scores for COPDGene (*p* < 0.001).

**Conclusion:**

Quantifying heterogeneous COPD manifestations as anomaly offers advantages over supervised methods and was found to be predictive for lung function impairment and morphology deterioration.

**Clinical relevance statement:**

Using deep learning, lung manifestations of COPD can be identified as deviations from normal-appearing chest CT and attributed an anomaly score which is consistent with decreased pulmonary function, emphysema, and air trapping.

**Key Points:**

• *A self-supervised DL anomaly detection method discriminated low-risk individuals and COPD subjects, outperforming classic DL methods on two datasets (COPDGene AUC = 84.3%, COSYCONET AUC = 76.3%).*

• *Our contrastive task exhibits robust performance even without the inclusion of expiratory images, while voxel-based methods demonstrate significant performance enhancement when incorporating expiratory images, in the COPDGene dataset.*

• *Anomaly scores improved the fitting of linear mixed effects models in predicting clinical parameters and imaging alterations (p < 0.001) and were directly associated with clinical outcomes (p < 0.001).*

**Supplementary Information:**

The online version contains supplementary material available at 10.1007/s00330-023-10540-3.

## Introduction

Chronic obstructive pulmonary disease (COPD) affects approximately 10.3% of the global population [1]. However, a significant portion of individuals remain undiagnosed [2], mainly due to the limitations of clinical tests and spirometry [3], in capturing the heterogeneous manifestations of the disease. These can range from predominant involvement of the airways to predominant damage and loss of the lung parenchyma. Quantitative CT imaging has emerged as a potential diagnostic tool [4], offering valuable insights into COPD manifestation [5, 6]. Nevertheless, the analysis and interpretation of CT images in these patients is challenging due to the variability and inhomogeneous distribution of findings related to COPD.

In recent years, supervised deep learning (DL) methods have been proposed to assist physicians in studying the various imaging characteristics of COPD [7–10]. Unfortunately, supervised approaches face limitations in capturing the full spectrum of COPD manifestations and representing them on the training data. In reality, this appears difficult, and typically results in poor generalizability. Additionally, these methods rely on obtaining local and global labels, which can be difficult and subjective to acquire. Moreover, previous DL methods have primarily focused on utilizing a single CT image during full inspiration, neglecting the potential benefits of incorporating full expiration CT scans as surrogate markers for small-airway inflammation [11, 12]. While large-scale cohort studies include expiratory CT scans for this purpose, their value for DL methods in COPD diagnosis has not yet been explored.

To address the limitations of existing supervised methods, this study used a self-supervised contrastive pretext model deep learning (DL) approach. Unlike supervised learning, self-supervised learning does not require labeling of pre-defined features. Instead, it leverages inherent patterns or relationships within the data, such as similarity between images, to create its own training labels and learn useful representations. Our hypothesis is that by leveraging the inherent similarities within normal-appearing lung regions and identifying deviations from these characteristics, COPD regions can be detected without explicitly learning all possible image features. By this approach, the spectrum of diseased lung parenchyma is implicitly captured as anomalies from what is found in healthy subjects (never-smoker controls) or patients with minimal disease (GOLD 0, no airflow limitation and no/or minimal emphysema) [13]. The presented study compares the proposed self-supervised anomaly detection approach with state-of-the-art supervised methods, explores the potential added value of incorporating expiratory CT scans under different input configurations, and contributes to a deeper understanding of the clinical implications in two nationwide cohorts.

## Materials and methods

### Study cohorts

Two multicenter cohorts were retrospectively used: COPDGene (Genetic Epidemiology of COPD) and COSYCONET (COPD and SYstemic consequences-COmorbidities NETwork). The COPDGene study (ClinicalTrials.gov Identifier: NCT00608764) [14] recruited current and former self-reported non-Hispanic whites and African Americans smokers (≥ 10 pack-years), aged 45–80 years, between 2008 and 2011. The COSYCONET imaging sub-study (ClinicalTrials.gov Identifier: NCT02629432) [15] recruited individuals with a diagnosis of COPD, according to the Global Initiative for Chronic Obstructive Lung Disease (GOLD) criteria [16], or chronic bronchitis, aged 40 years or older, between 2010 and 2013. Both studies collected paired chest CT in inspiration (Insp) and expiration (Exp), pulmonary function tests (PFT), and questionnaires. Reconstruction protocols were comparable, but the maximum dose level for the CT acquisition differed (3.5 mSv for COSYCONET and 10 mSv for COPDGene, respectively). For additional details on the CT protocol, see Supplementary S1.

To account for disparities in health outcomes and quality of life between African Americans and non-Hispanic whites with COPD [17–19], a self-reported non-Hispanic white population was conveniently selected from COPDGene to match the ethnicity of COSYCONET.

**Fig. 1:**
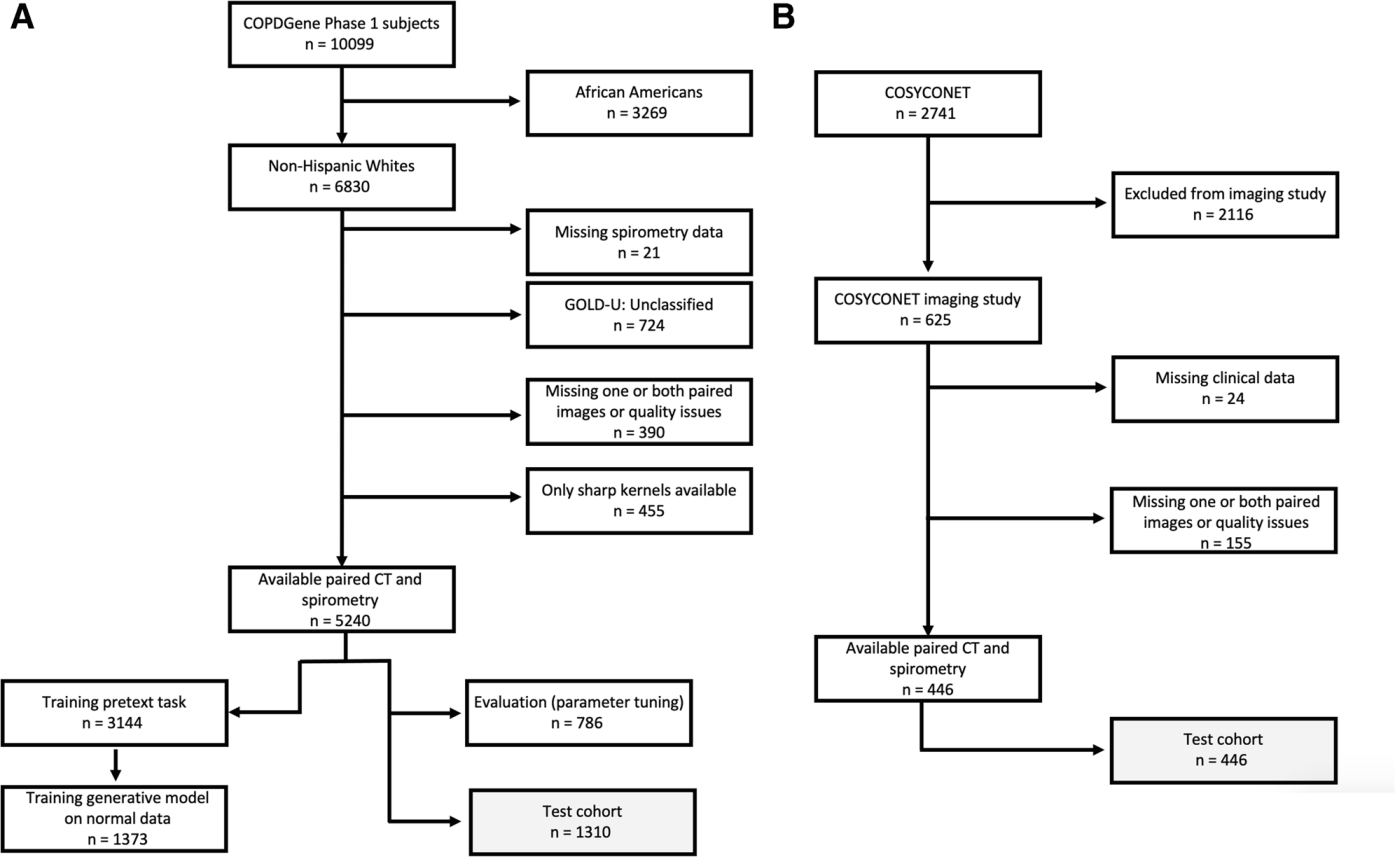
Study design flowcharts for data selection. **A** Data selection from the COPDGene cohort. Data from 3144 participants were used to train the pretext task, from which 1373 normal never-smoker control and GOLD 0 subjects were used to train the generative model. The evaluation task (hyperparameter tuning) was performed on 786 subjects. The final testing was performed only once on the test set (1310 subjects). **B** Data selection from the COSYCONET cohort. This was entirely used as an external test set (*n* = 446)

Exclusion criteria and study design are shown in Fig. 1A and B.

The “control” class was defined as individuals without airflow obstruction, as indicated by forced expiratory volume in 1 s (FEV_1_) to forced vital capacity (FVC) ratio equal to or greater than 70%. This category encompassed never-smoker controls and GOLD 0 individuals, none of whom met the full criteria for a COPD diagnosis. As defined by the inclusion criteria to the cohort studies, these individuals did not present other lung diseases.

The “diseased” class was defined by individuals with clinical manifestations of COPD and airflow obstruction (FEV_1_/FVC < 70%), corresponding to GOLD 1–4.

Written consent was obtained, and the study protocol was approved by each clinical center’s review board.

### Pre-processing

The input for the DL model were 3D ROIs (patches) extracted from single Insp scans or from dual-channel Insp and registered Exp (ExpR) CT scans, respectively, covering > 70% of the segmented lung parenchyma volume of each individual. The chosen patch size of 50^3^ voxels (50 × 50 × 50 voxels) was meant to cover the typical size of a secondary pulmonary lobule, the basic unit of lung structure [20]. Two patch-overlapping strategies were implemented (0% and 20%) and applied to Insp CT (1 channel) and Insp + ExpR (2 channels), resulting in four different configurations of input patches to be tested.

To ensure that patches extracted from the “control” class individuals were representative of healthy lung regions, only those with less than 1% emphysema were included when acquiring the representative distribution model of normal-appearing lung. Further pre-processing details are found in Supplementary S2 and Supplementary Figure 1.

### COPD Classification as Out-Of-Distribution anomaly detection (cOOpD)

**Fig. 2:**
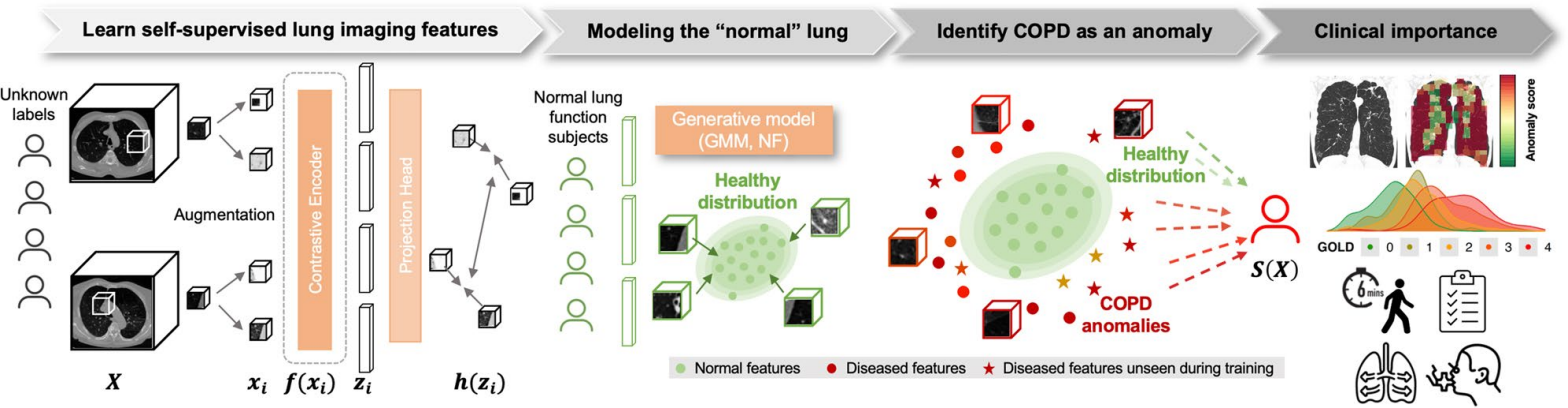
Overview of the pipeline. Preprocessed 3D patches *x*_*i*_ are extracted from the lung parenchyma from Insp and ExpR CT images *X*. Informative lung representations *z*_*i*_ at the patch level are learned through a self-supervised contrastive pretext task *f(x*_*i*_*)*, based on the rationale of clustering together unlabeled positive pairs while pushing apart negative pairs. A generative model based on a Gaussian mixture model (GMM) or normalizing flow (NF) is then applied on the representations from normal regions of “control” individuals, so that the normal patient lung can be modeled. From this healthy distribution, anomalies/deviations are then detected as COPD-like regions. An anomaly score is assigned per patch based on the negative log likelihood. Patient-level predictions *S(X)* are obtained by aggregating the anomaly scores from all patches of the lung. Clinical meaning of these scores can be derived with anomaly maps. Further, patient score distribution can be correlated to severity stages and pulmonary function tests, as the St. George’s Respiratory Questionnaire, 6-min walking test, modified Medical Research Council dyspnea score, and spirometry

A sequence of *B* 3D lung patches ${\left\{x_i\right\}}_{i=1}^{B}$ is taken per patient *i*, from a single or paired CT scan *X*. A latent representation is obtained per each patch, for a maximum of 100 patches per subject selected at random, using a trained self-supervised contrastive encoder *z*_*i*_* = f(x*_*i*_*)*. The maximum of 100 patches per subject was defined based on previous experiments, showing that using all patches available per subject only introduces redundancy while increasing computational costs. Then, the distribution of normal representations from all patches of control individuals is learned through a generative model *p(z)* and anomalies (assumed as stemming from COPD) are identified and given an anomaly score defined as the negative log likelihood *s(x*_*i*_*) = − log(p(f(x*_*i*_*)))*. A patient-level score *S(X)* is obtained by aggregating all patch-level scores, which is then used to predict the binary class (“control” vs “diseased” classes). Final aggregation strategy was chosen based on the highest area under receiver operator curve (AUC) on three runs on the validation set, for all the input configurations (Insp 0%, Insp 20%, InspExpR 0%, InspExpR 20%). These steps are detailed in [13] and Supplementary S3–5 and presented in Fig. 2.

### Compared methods

Four established supervised deep learning methods, detailed in Supplementary S-6, were compared to cOOpD: three voxel-based (end-to-end Patch Classifier with a recurrent neural network [PatClass + RNN], a multiple instance learning [MIL] with RNN as aggregation [MIL + RNN], an attention-based MIL [MIL + Att]) and one representation-based [ReContrastive]).

### Statistical analysis and clinical prediction

The main evaluation metric for the COPD binary classification was the AUC (more details in Supplementary S-7).

For exploratory purposes, a two-way ANOVA was performed independently per dataset to evaluate the effect of the method, input configuration, and their interaction on the main performance metric. A post hoc Tukey test was then performed to assess the multiple pairwise comparisons in between each method and input configuration.

Linear mixed effects models (LMM) were used to predict clinical parameters and radiological features based on the cOOpD anomaly score (*S(X)*), adjusted for age, gender, body mass index (BMI), smoking status (0: never-smoker control, 1: former smoker, and 2: current smoker), smoking duration, and a random term for the study site. Clinical parameters included the following: St. George’s Respiratory Questionnaire (SGRQ), 6-min walking test (6MWT), FEV_1_, or FEV_1_/FVC. Radiological features were the %Emphysema or the %Air Trapping, measured by Vida Diagnostics (Coralville, IA) (for COPDGene) and by YACTA [21–23] (for COSYCONET). As these radiological features were skewed, a log transformation was employed. We report the overall conditional coefficient of determination (*R*^2^), adjusted for the number of regressors, and the individual *R*^2^ decomposition of the explained variation. Forest plots present the standardized beta coefficients (estimates) of the fixed terms, per model. 95% confidence intervals (CIs) and *p*-values were computed using a Wald *t* distribution approximation. Added value of the anomaly score, compared to nested baseline methods, was assessed through the likelihood ratio test of nested models. Additionally, to test whether the anomaly score provides additional information beyond morphological lung changes, models were also adjusted for %Emphysema and %Air Trapping, for the prediction of PFT.

COPD outcomes prediction by the anomaly score was also analyzed, namely reported severe exacerbations in the past year (Wilcoxon rank-sum test) and dyspnea, by the modified Medical Research Council (mMRC) dyspnea scale (Jonckheere-Terpstra test).

Statistical analyses were performed with R (version 4.2.3; R Foundation for Statistical Computing). A *p*-value of < .05 was considered statistically significant and was adjusted for multiple comparisons, using the Holm-Bonferroni method.

The applied code is available on a public repository on GitHub (https://github.com/MIC-DKFZ/cOOpD).

## Results

### Clinical characteristics

Table 1 presents demographic data, functional parameters, and radiological measures for the training and test sets. The self-supervised pretext task training cohort consisted of 3144 COPDGene participants, of which 63 never-smokers and 1310 GOLD 0 met the criteria for the “control” class, as defined above. Healthy-appearing samples from this group were used to acquire the representative distribution model of the “control” lung. The average percentages of emphysema and air trapping in the entire lungs of these subjects were 2.5% and 11.7%, respectively. This was considered to be consistent with low-risk and control groups from previously published studies [24–26].

**Table 1:** Demographic data, functional parameters, and low-attenuation (LAA) percentages, for the pretext self-supervised contrastive task, for the generative model on the “control” class from the COPDGene dataset, and for the internal (COPDGene) and external test set (COSYCONET). Note: Attenuation percentages were measured by different methods: VIDA Diagnostics for COPDGene, YACTA for COSYCONET

Characteristic	Training pretext task (COPDGene) (“normal” and “diseased” class)	Training generative model (COPDGene) (only “normal” class)	Internal test set (COPDGene) (“normal” and “diseased” classes)	External test set (COSYCONET) (“normal” and “diseased” classes)
Demographic data				
*N* of patients [*N*]	3144	1373	1310	446
M [*N*]	1699	694	692	272
F [*N*]	1445	679	618	174
Age (y) [mean, (IQR)]	63 (55–69)	60 (52–66)	63 (56–69)	63 (58–69)
BMI [mean, (SD)] ^†^	28.3 (5.7)	29.1 (5.6)	28.1 (5.7)	26.6 (4.6)
Smoking habits				
Never-smoker [*N*, (%)]	63 (2.0%)	63 (4.6%)	29 (2.2%)	34 (7.6%)
Former smoker [*N*, (%)]	1862 (59.2%)	755 (54.9%)	817 (62.4%)	298 (66.7%)
Current smokers [*N*, (%)]	1219 (38.8%)	555 (40.4%)	464 (35.4%)	114 (25.6%)
Smoking duration (y) [mean, (SD)] ^†^	36 (12)	31 (13)	36 (12)	33 (15)
Spirometry				
FEV_1_%_pred [mean, (SD)]	75.4 (26.8)	97.2 (11.3)	74.2 (27.1)	57.6 (19.1)
FEV_1_/FVC [mean, (SD)]	0.6 (0.2)	0.8 (0.1)	0.6 (0.2)	0.7 (0.2)
Non-smoker control [*N*]	63	63	29	0
GOLD 0 [*N*]	1310	1310	538	23
GOLD 1 [*N*]	350	0	128	30
GOLD 2 [*N*]	764	0	315	215
GOLD 3 [*N*]	423	0	195	146
GOLD 4 [*N*]	234	0	105	32
Severe exacerbations [*N* (%)]	318 (10.1%)	40 (3%)	137 (10.5%)	224 (50.3%)
6MWT (ft) [mean, (SD)] ^*+#†^	433.8 (120.7)	485.4 (98.6)	427.1 (124.8)	453.3 (99.8)
SGRQ [mean, (SD)] ^†^	25.6 (22.5)	13.3 (15.5)	25.9 (22.9)	40.3 (18.8)
mMRC dyspnea score 0 [*N*] ^*#†^	1502	963	588	274
mMRC dyspnea score 1 [*N*] ^*#†^	460	193	221	110
mMRC dyspnea score 2 [*N*] ^*#†^	364	104	158	60
mMRC dyspnea score 3 [*N*] ^*#†^	549	98	225	1
mMRC dyspnea score 4 [*N*] ^*#†^	264	15	115	0
Imaging				
Imaging (Insp and Exp)				
LAA-950%* [mean, (SD)] ^*+#^	7.8 (10.4)	2.5 (3.0)	8.1 (10.6)	17.0 (13.6)
LAA-856%* [mean, (SD)] ^*+#^	25.4 (20.4)	11.7 (9.9)	26.1 (21.1)	45.2 (20.5)

*COPDGene*, Genetic Epidemiology of COPD; *COSYCONET*, COPD and SYstemic consequences-COmorbidities NETwork;* N*, number; *SD*, standard deviation; *y*, years; *BMI*, body mass index; *FEV*_*1*_, forced expiratory volume in 1 s; *FEV*_*1*_*/FVC*, FEV_1_-to-forced vital capacity ratio; *GOLD*, Global Initiative for Chronic Obstructive Lung Disease; *6MWT*, 6-min walking test; *SGRQ*, St. George’s Respiratory Questionnaire; *LAA-950%*, percentage of LAA under − 950 HU; *LAA-856%*, percentage of LAA under − 856 HU

For some cases, data was not available:

^*^(48 for 6MWT, 5 for mMRC dyspnea score, 7 for LAA-950%, 163 for LAA-856%);

^+^(7 for 6MWT, 3 for LAA-950%, 65 for LAA-856%);

^#^(17 for 6MWT, 3 for mMRC dyspnea score, 3 for LAA-950%, 76 for LAA-856%);

^†^(1 for BMI, 3 smoking duration, 22 for 6MWT, 2 for SGRQ, 1 for mMRC dyspnea score)

The internal testing cohort consisted of 1310 COPDGene participants (692 men, 618 women), with mean age 63 years (interquartile range [IQR] 56–69). The external testing cohort consisted of 446 COSYCONET participants (272 men, 174 women), with mean age 63 years (IQR 58–69).

### COPD binary classification and effect of including the expiratory CT

**Fig. 3:**
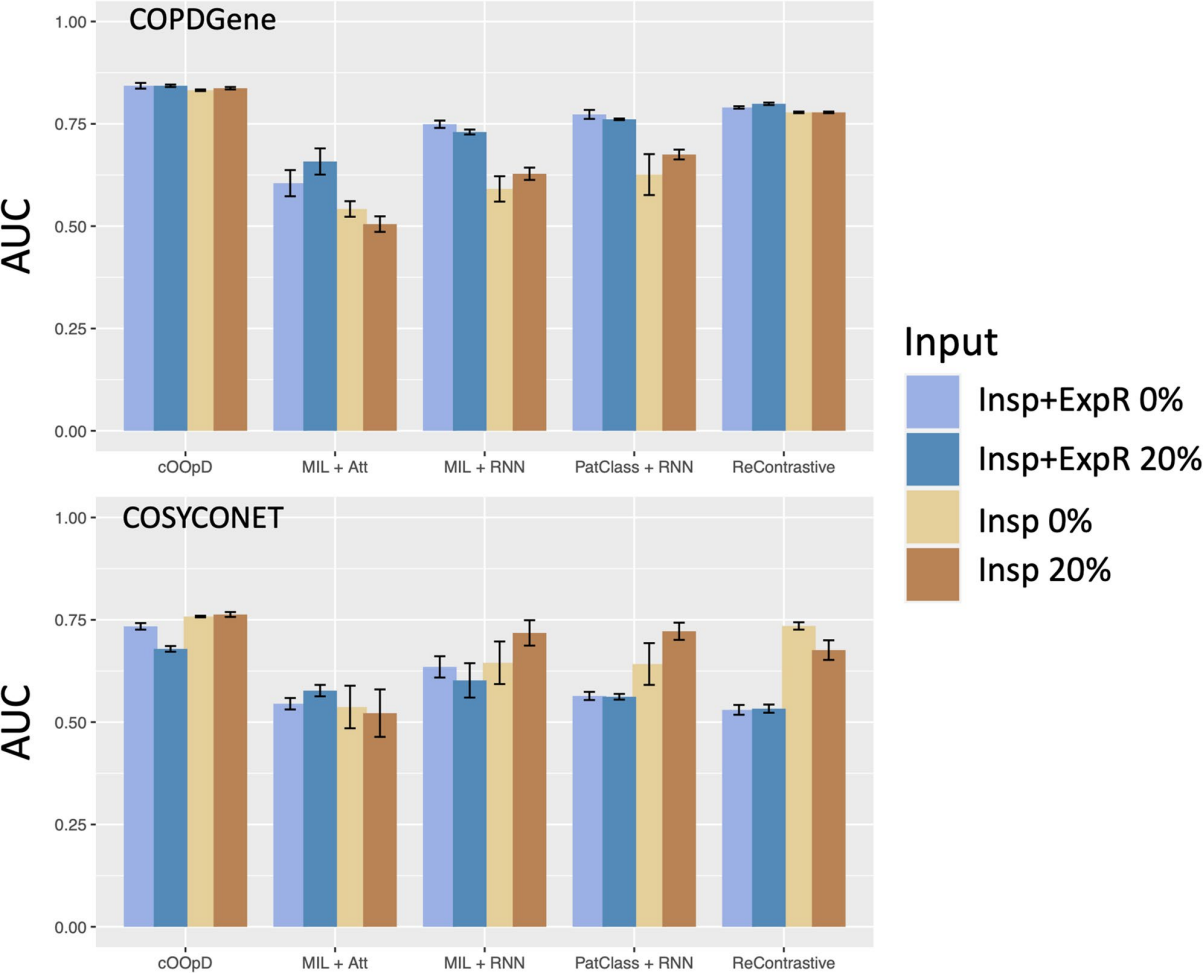
Performance assessment through the area under the receiver operating curve (AUC) for the internal (COPDGene) and external (COSYCONET) test sets, for four different input configurations [0% and 20% patch-overlapping applied to Insp CT (1 channel) and Insp + ExpR (2 channels)], for the anomaly detection method (cOOpD), and for four supervised deep learning methods: end-to-end Patch Classifier with a recurrent neural network [PatClass + RNN], a multiple instance learning [MIL] with RNN as aggregation [MIL + RNN], an attention-based MIL [MIL + Att], and one representation-based [ReContrastive]

The average performances of all models and all input configurations on the internal and external test sets (Fig. 3) were examined. On COPDGene, the best average performance was achieved by our proposed model (cOOpD), reaching an AUC of 83.2 ± 0.2, 83.7 ± 0.3, 84.3 ± 0.7, and 84.3 ± 0.3 for Insp-0%, Insp-20%, Insp + ExpR-0%, and Insp + ExpR-20%, respectively. All methods showed a slightly lower performance on the external test set (COSYCONET), but the proposed method remained the most performant, achieving an AUC of 75.8 ± 0.2, 76.3 ± 0.6, 73.4 ± 0.8, and 67.9 ± 0.7 for Insp-0%, Insp-20%, Insp + ExpR-0%, and Insp + ExpR-20%, respectively (Supplementary Table 3).

For exploratory purposes, the effect of method, input configuration, and their interaction for overall performance was evaluated (Table 2). For both datasets (COPDGene and COSYCONET), we found a statistically significant difference in the average AUC by the method, the input configuration, and their interaction.

**Table 2:** Two-way analysis of variance (ANOVA) per dataset (COPDGene and COSYCONET) to evaluate the effect of the method, input configuration, and their interaction on the main performance metric (AUC). The second column presents the *F* statistics, where the number in parentheses corresponds to the degrees of freedom (*N* – 1)

Source of variation	*F* statistics	*p*-value
COPDGene		
Method	*F*(4) = 393.94	*p* < 0.001
Input configuration	*F*(3) = 94.43	*p* < 0.001
Method:input configuration (interaction)	*F*(12) = 14.36	*p* < 0.001
COSYCONET		
Method	*F*(4) = 50.43	*p* < 0.001
Input configuration	*F*(3) = 24.00	*p* < 0.001
Method:input configuration (interaction)	*F*(12) = 7.46	*p* < 0.001

*COPDGene*, Genetic Epidemiology of COPD; *COSYCONET*, COPD and SYstemic consequences-COmorbidities NETwork; *N*, number of variables

The Tukey post hoc test revealed that, for both test sets, our proposed method yielded, on average, higher AUC scores than all other methods (*p* < 0.001), regardless of the input configuration.

The effect of adding the ExpR, on the other hand, only showed, on average, statistically significant improvements for the voxel-wise supervised methods (MIL + Att, MIL + RNN, PatClass + RNN) for COPDGene (*p* < 0.001). When applied to the data from COSYCONET, this effect was no longer observed.

The subsequent analyses were conducted for the configuration that achieved higher mean AUC and lower standard deviation (InspExpR-20%).

### Visualization of anomaly maps

**Fig. 4:**
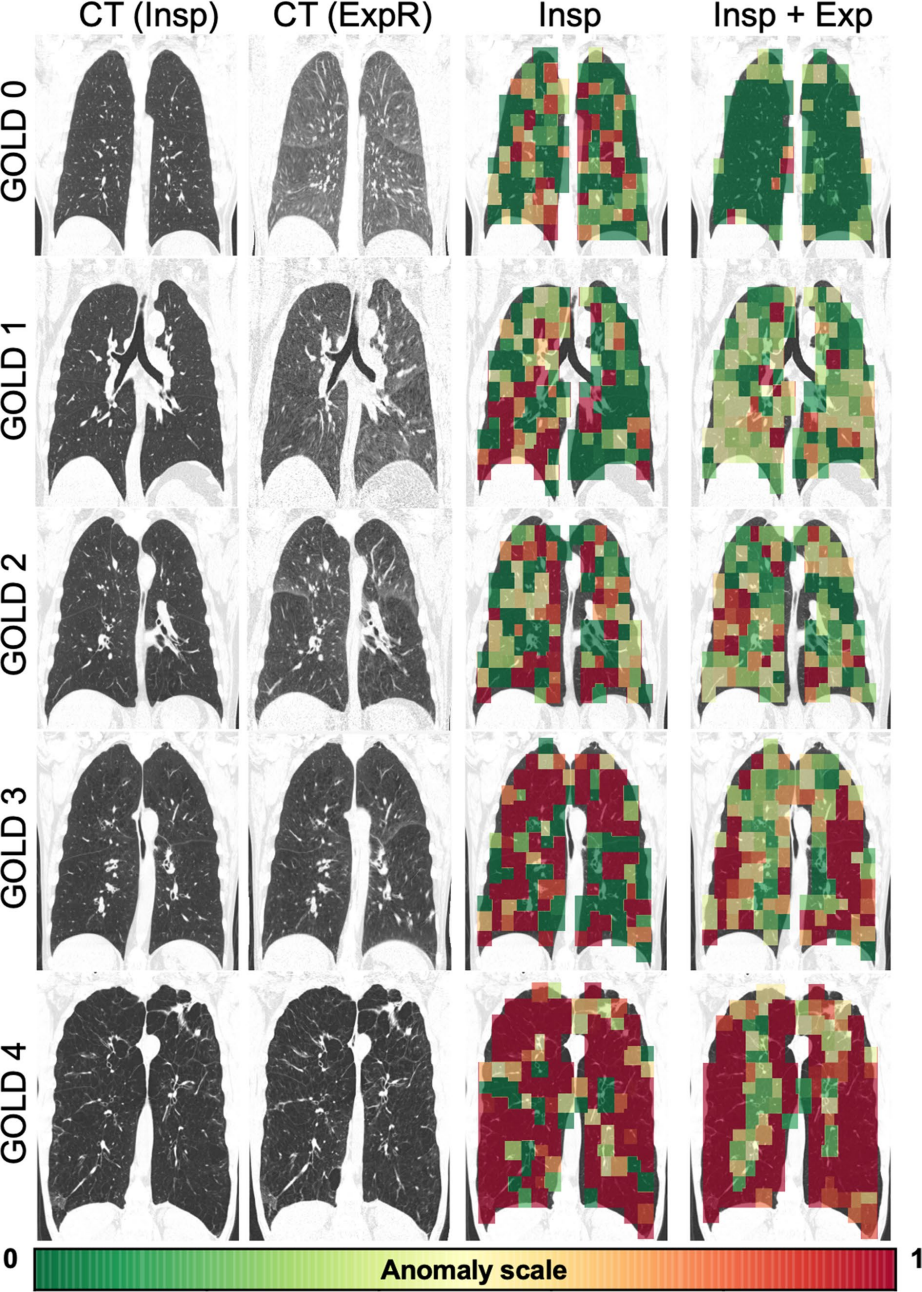
Representative coronal views of the cOOpD score map using the inspiratory image alone (3rd column) and using the Insp and the ExpR image (4th column) on five subjects from COPDGene with different degrees of severity, for a 20% patch-overlapping strategy. CT images are scaled from − 1300 to 50 HU for visualization purposes. Color maps show the normalized patch anomaly score (negative log likelihood), normalized by the min–max normalization corresponding to the 5th and 95th percentiles of the dataset. Red symbolizes higher degrees of severity

Figure 4 shows representative coronal CT views with an overlay of the patch-level anomaly scores obtained for InspExpR-20% and Insp-20% (as reference), which illustrate how much certain lung regions differentiate from normal-appearing ones. Min–max normalization was applied for visualization purposes, corresponding to the 5th and 95th percentiles of the corresponding test set.

### Clinical and radiological predictive value: COPDGene

Adding the anomaly score statistically improved the fitting of all LMM to predict clinical and radiological measures (*p* < 0.001 each) (Table 3). Similar models adjusted for %Emphysema, or adjusted for the %Air Trapping, to predict PFT, also benefit from adding the anomaly score as predictor (6MWT, FEV_1_, FEV_1_/FVC, *p* < 0.001 for each). This indicates that the anomaly score comprises more than the morphological information provided by %Emphysema or %Air Trapping.

**Table 3:** Linear mixed effects model to predict several clinical (SGRQ, 6MWT, FEV_1_, FEV_1_/FVC) and radiological (%Emphysema, %Air Trapping) dependent variables using the produced anomaly score as a predictor, for the COPDGene test cohort (*n* = 1310). Note: Models are adjusted for age, gender, BMI, smoking status, smoking duration, and a random term for the study site. This model was compared with a baseline model that omits the anomaly score. Conditional *R*^2^ is adjusted for the number of regressors added. Bold values indicate a greater R^2^ per dependent variable. *p*-values are reported per model and for the comparison between them and are corrected for multiple comparisons. * %Emphysema and %Air Trapping were skewed, so a log transformation was applied

Dependent variable	Predictor	Adjusted conditional *R*^2^	*p*-value	
SGRQ	Age, gender, BMI, smoking status, smoking duration, (center)	0.24	*p* < .001	*p* < .001
	Age, gender, BMI, smoking status, smoking duration, anomaly score, (center)	**0.36**	*p* < .001	
6MWT	Age, gender, BMI, smoking status, smoking duration, (center)	0.41	*p* < .001	*p* < .001
	Age, gender, BMI, smoking status, smoking duration, anomaly score, (center)	**0.45**	*p* < .001	
FEV_1_	Age, gender, BMI, smoking status, smoking duration, (center)	0.22	*p* < .001	*p* < .001
	Age, gender, BMI, smoking status, smoking duration, anomaly score, (center)	**0.49**	*p* < .001	
FEV_1_/FVC	Age, gender, BMI, smoking status, smoking duration, (center)	0.26	*p* < .001	*p* < .001
	Age, gender, BMI, smoking status, smoking duration, anomaly score, (center)	**0.54**	*p* < .001	
Emphysema % *	Age, gender, BMI, smoking status, smoking duration, (center)	0.38	*p* < .001	*p* < .001
	Age, gender, BMI, smoking status, smoking duration, anomaly score, (center)	**0.54**	*p* < .001	
Air Trapping % *	Age, gender, BMI, smoking status, smoking duration, (center)	0.33	*p* < .001	*p* < .001
	Age, gender, BMI, smoking status, smoking duration, anomaly score, (center)	**0.58**	*p* < .001	

*R*^*2*^, overall conditional coefficient of determination; *BMI*, body mass index; *SGRQ*, St. George’s Respiratory Questionnaire; *6MWT*, 6-min walking test; *FEV*_*1*_, forced expiratory volume in 1 s; *FEV*_*1*_*/FVC*, FEV_1_-to-forced vital capacity ratio

Furthermore, the explanatory power (*R*^2^) of all models increased by adding the anomaly score, with the most significant improvement observed in predicting radiological features. Compared to other predictors, the anomaly score had the highest individual explained variance for all models (Supplementary Table 4). In detail, it explained 19% (95%CI 16, 25), 12% (95%CI 8, 15), 39% (95%CI 35, 44), 41% (95%CI 38, 45), 28% (95%CI 23, 32), and 40% (95%CI 37, 45) of the variance of the SGRQ, 6MWT, FEV_1_, FEV_1_/FVC, %Emphysema, and %Air Trapping, respectively.

**Fig. 5:**
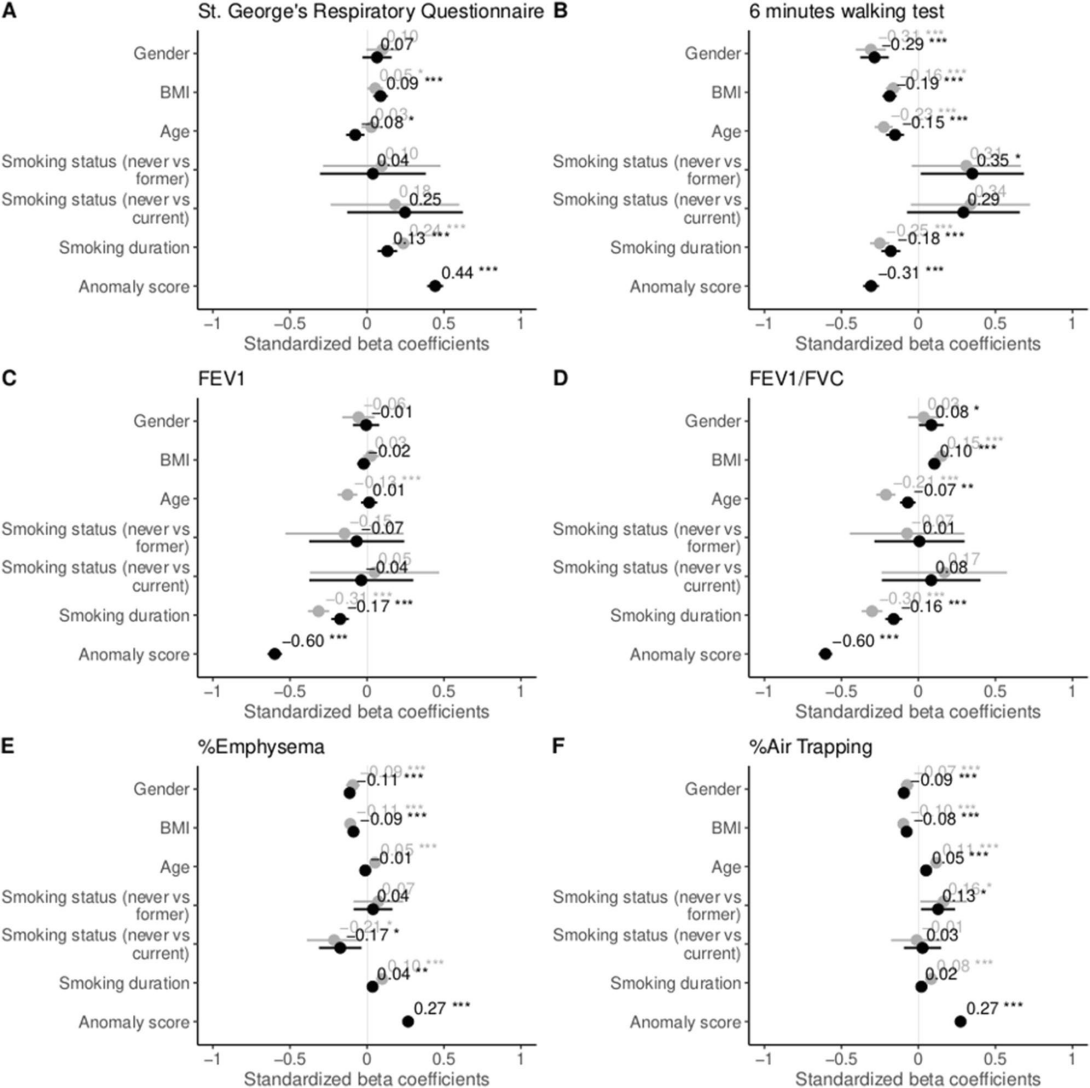
COPDGene forest plots of standardized beta values of the linear mixed effects models to predict the St. George’s Respiratory Questionnaire (**A**), 6-min walking test (**B**), FEV_1_ (**C**), FEV_1_/FVC (**D**), %Emphysema (**E**), and %Air Trapping (**F**). %Emphysema and %Air Trapping were skewed, so a log transformation was applied. Baseline models (without the anomaly score) are colored in gray, while baseline + anomaly score models are colored in black (*p* < 0.05*/0.01**/0.001***). BMI, body mass index; FEV_1_, forced expiratory volume in 1 s; FEV_1_/FVC, FEV_1_-to-forced vital capacity ratio

As shown in Fig. 5, BMI, smoking duration, and the anomaly score were positively correlated with predicting SGQR. An increase in one standard deviation (SD) of the anomaly score resulted in a 0.44 increase of the SD of SGRQ. For the 6MWT, the lower the BMI, age, smoking duration, and anomaly score, the higher the distance a patient can walk. This distance was lower for a female patient than for a male patient. Here, for an increase in one SD of the anomaly score, the SD of the distance a patient can walk in 6 min decreased 0.31 times. For FEV_1_, both the effect of longer smoking duration and higher anomaly scores were significantly correlated with a low FEV_1_. No statistically significant differences were found for gender or BMI. For FEV_1_/FVC, the trends were similar, except for BMI, which was positively correlated. For both spirometry measures, an increase in one SD of the anomaly score was associated with a decrease of 0.60 of the SD of FEV_1_ or FEV_1_/FVC, almost four times more than for a unit decrease in the SD of smoking duration.

**Fig. 6:**
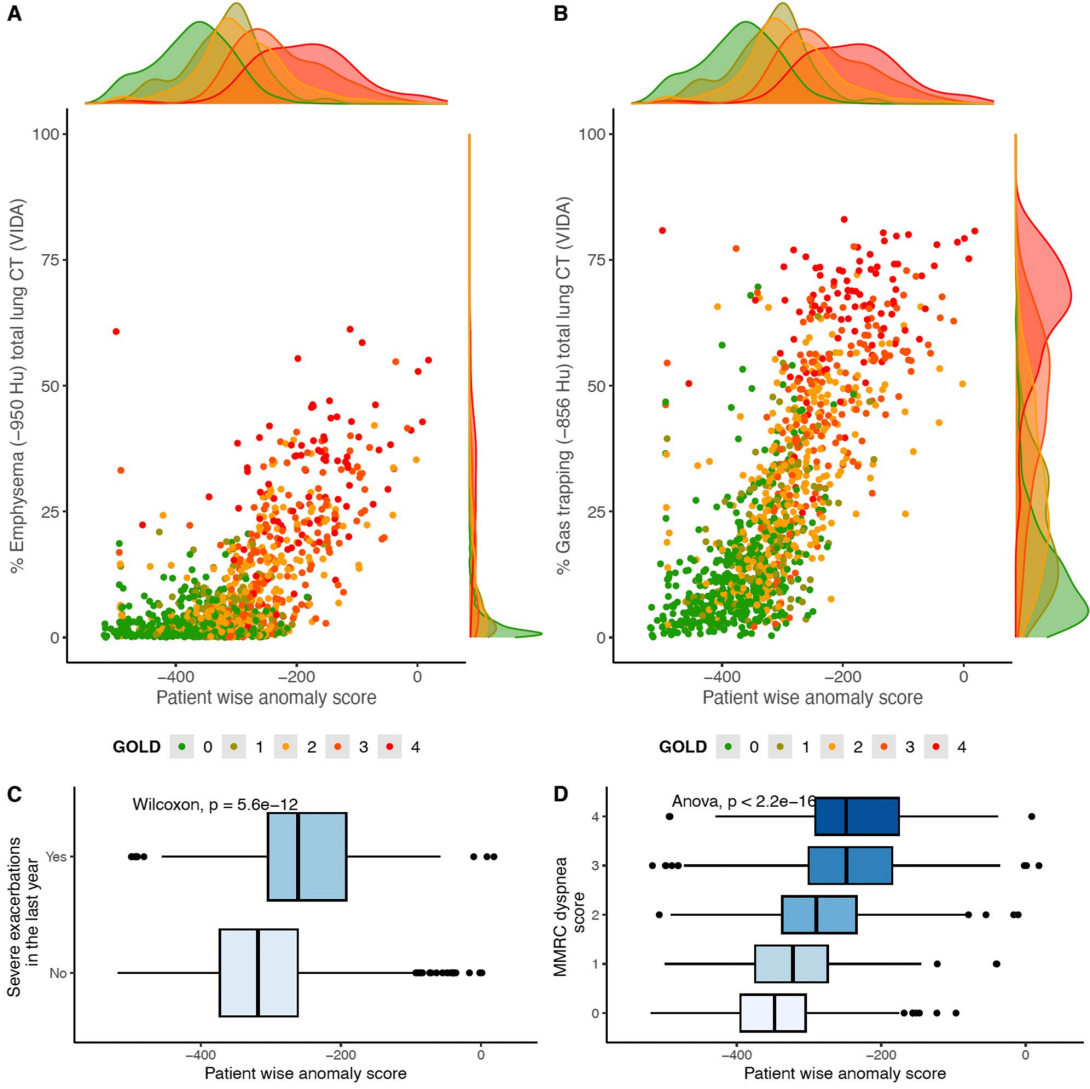
Distribution of patient-wise anomaly scores from COPDGene versus %Emphysema (**A**) and %Air Trapping (**B**), with reported Pearson’s correlation coefficient (*r*), colored by GOLD stage. Subjects who experienced severe exacerbations in the past year were significantly different than those who did not in terms of the anomaly score (*p* < 0.001, Wilcoxon test) (**C**). The distribution of the anomaly score differed among the modified Medical Research Council (mMRC) dyspnea classes (*p* < 0.001, Jonckheere-Terpstra) (**D**)

For radiological features, lower BMI and higher anomaly scores were significantly correlated with higher %Emphysema and %Air Trapping. Both measures were significantly lower for female patients than for male patients. An increase in one SD of the anomaly score was associated with an increase of 0.27 SD for %Emphysema and %Air Trapping. This relationship is further highlighted in Fig. 6A and B, where the distributions of the anomaly score and the %Emphysema and %Air Trapping are shown. The anomaly score density plots (top) colored by the GOLD stage show a distinction between the GOLD classes, especially for GOLD 0 and 4. This distinction is no longer clear for the %Emphysema density plots, as the GOLD classes are highly overlapped.

Finally, the anomaly score was associated with worsening of COPD symptoms. Patients who reported severe exacerbations in the past year had a higher anomaly score (*p* < 0.001) (Fig. 6C). Increases in dyspnea scores were also associated with higher anomaly scores (*p* < 0.001) (Fig. 6D).

### Clinical and radiological predictive value: COSYCONET

Adding the anomaly score statistically improved the fitting of all LMM models (*p* < 0.001 each), except for SGRQ. The explanatory power followed the same trends as in COPDGene, although less strong (Supplementary Table 5). Individual explained variance of the predictors (Supplementary Table 6) and standardized beta forest plots (Supplementary Figure 2) were consistent with the results reported before: higher anomaly scores were associated with greater SGRQ, more severe emphysema and air trapping (Supplementary Figure 3A, B), and lower walking distances and lung function decline. Again, patients who reported severe exacerbations in the past year had higher anomaly scores (*p* < 0.001) but increases in dyspnea scores were no longer associated with higher anomaly scores (Supplementary Figure 3C, D).

## Discussion

In this study, we reformulate COPD binary classification into an anomaly detection task. We leverage the heterogeneity of COPD by modeling characteristics of “normal” lung tissue from low-risk “control” individuals and identifying deviations as anomalies indicative of impaired regions. Our method, based on self-supervised contrastive deep learning, outperformed supervised classification models in two cohorts: COPDGene (AUC 84.3 ± 0.3, *p* < 0.001) and COSYCONET (AUC 76.3 ± 0.6, *p* < 0.001). The anomaly detection task was trained solely on differentiating COPD patients from healthy individuals and individuals with minimal disease and minimal emphysema and without airflow limitation (GOLD 0), which is basically a binary decision. However, even without ever giving the disease severity information (e.g., GOLD classification) to the model during training, it was able to produce an anomaly score, which is quantitatively consistent with a wide range of clinical variability, including symptoms, spirometry, and imaging findings associated with COPD.

Clinically, our study has three important implications.

Firstly, we demonstrate that our proposed contrastive task appears to be robust even if expiratory images were not included. The additional value was quantified as the difference in performance metrics, contrasting this with voxel-based methods, which heavily rely on labeled data and showed a significant improvement for COPDGene when expiratory images were incorporated. As a possible explanation for the robustness of our contrastive task model even when limited to inspiratory images alone instead of using both, in- and expiratory images, one could have discussed an already high prevalence of small airway disease and consecutive findings on expiratory CT images (i.e., air trapping) in the minimal risk group (defined as “control” for the purposes of this study). This could have reduced the difference to the “diseased” group, in particular for features related to expiratory CT images. However, this appears unlikely, since in the above defined “control” class, which we employed to model the normal lung distribution, mean prevalence of emphysema and air trapping across the total lung volume were 5.5% and 11.7%, respectively. These findings are in line with expected clinical imaging characteristics for a healthy lung at their respective ages. In a COPD study, Lv et al [26] reported mean emphysema percentage of 5.92% and an air trapping percentage of 14.32% in a cohort of 86 low-risk individuals with FEV_1_/FVC ≥ 80%. Similarly, in a study from asthmatics, Busacker et al [25] reported an air trapping percentage of 12.3% in 26 healthy controls (normal lung function and negative methacholine bronchoprovocation). Our findings closely align with these established benchmarks for normal lung characteristics. A relevant bias related to probable minimal findings, i.e., on expiratory CT of the minimal-risk group (“control”), which may reduce the efficacy of our self-supervised contrastive deep learning methodology in separating the two groups therefore appears unlikely. Further proof is provided by the fact that improvements of model performance by including expiratory CT scans were indeed observed for the voxel-based DL models, which were run for comparison.

Secondly, the anomaly score maps generated per subject allow for the immediate identification of abnormal regions. This might be clinically useful, potentially serving as an alert tool in case finding scenarios that can be further examined by radiologists.

Thirdly, patient-wise anomaly scores may serve as clinical surrogate markers or biomarkers, providing valuable insights into the heterogeneity of COPD. We show that these scores predict clinical and radiological features, enhancing the understanding of their real meaning and improving the fitting of linear mixed effects models for parameters such as walking distance, respiratory function, %Emphysema, and %Air Trapping (*p* < 0.001). Furthermore, higher anomaly scores are associated with severe exacerbations, dyspnea scores, and other disease-related factors (*p* < 0.001). Further research is warranted to assess the clinical value of anomaly scores for the detection and quantitative assessment of disease exacerbation on the one hand and on following up disease manifestation in individual patients on the other hand.

It is known that the correlation between morphological changes and PFTs is limited [27]. We have shown that adding the anomaly score to models adjusted for %Emphysema or %Air Trapping, in the prediction of PFTs, is advantageous (*p* < 0.001), for both standard-dose (COPDGene) and low-dose scenarios (COSYCONET). We hypothesize that the anomaly score incorporates information beyond morphology, overcoming the limitation of PFTs being impaired by further underlying diseases, such as bronchial obstruction.

To our knowledge, no other work has developed a patient- or region-wise anomaly score that could quantify deviations from typical healthy subjects. Most studies focus on leveraging deep learning features for binary classification of chest CT. For instance, González et al [7] achieved an AUC of 85.6% using a 2D-CNN on COPDGene, while Tang et al [8] improved it to 88.9% using a more complex network and multi-channel slices on PanCan. Singla et al [9] proposed an auto-encoder with attention for COPD outcome prediction (AUC 82%) and suggested saliency maps for explainability. Sun et al [10] used a MIL + Att strategy to detect COPD (AUC 93.4%) and categorize GOLD stages, but lacked correlation with clinical information, and the dataset was not publicly available. Recently, Park et al [28] developed a DL model to predict pulmonary function using low-dose CT scans, achieving concordance correlation coefficients of 0.91 for FEV_1_. However, these were individuals from health screening services, mostly without respiratory disease. When evaluated solely at the high-risk group (FEV_1_/FVC < 0.70), the model revealed fairly low sensitivity (61.6%). Li et al [29] included voxel-to-voxel matching in their DL model, identifying seven “factors” in the SPIROMICS dataset. F0 and F4 were highlighted as surrogate markers for local abnormalities in healthy-risk and GOLD3-GOLD4, respectively, and regression models explained 59% of the variance of FEV_1_ and 66% of FEV_1_/FVC. F0 and F4 were positively correlated with %Emphysema (*r* = 0.54, *r* = 0.51) and Air Trapping% (*r *= 0.73, 0.56). Our results are very much in line with previously reported ones, particularly with regressions from Li et al (COPDGene: *R*^2^ = 49% and *R*^2^ = 54% for FEV_1_, FEV_1_/FVC; *r* = 0.65 and *r* = 0.74 for %Emphysema and % Air trapping).

The presented approach differs from the aforementioned studies by leveraging the inherent heterogeneity of COPD, by detecting anomalies from the “control” group. Instead of relying on supervised methods which require ample training data [7–10, 28], or unsupervised methods that necessitate selecting specific factors and embeddings [29], we adopt a simple agnostic self-supervised DL approach. Both of the best performing methods (cOOpD and ReContrastive) employed self-supervised DL. In consistence with previous works [13, 30], this supports the hypothesis of better performances when moving from voxels to representations, due to its enriching information and lower dimensionality. Furthermore, modeling the imaging traits (i.e., self-supervised representations) from the COPDGene “control” group appeared to be a suitable foundation for detecting abnormalities in patient scans and for improvement of generalizability of this approach. As expected, the performance dropped on the test data set from COSYCONET, but anomaly scores showed the same associations to clinical and radiological features as seen in the COPDGene test set. High class imbalance, different cohort inclusion criteria, and differences in CT protocols, namely dose differences, may be the origin of this variation. Still, anomaly scores showed the same associations to clinical and radiological features as seen in the COPDGene test set, which may be considered suggestive for the generalizability of this approach.

Some limitations of our study need to be discussed: Although we circumvent the need to label all the data, our method still requires defining a population of normal individuals, which in our study consisted of healthy never-smoker individuals and individuals with minimal disease and minimal emphysema and without airflow limitation (GOLD 0). As a measure to reduce potential bias from this, we only included patches with less than 1% emphysema to exclude regions with a certain degree of lung damage. Furthermore, it is worth noting that assembling a truly healthy population, comparable in size to the diseased cases in the COPDGene training dataset, poses significant challenges. This is particularly true in the context of our study, which necessitated paired inspiratory and expiratory CT scans. Such scans involve a radiation burden that may not be ethically justifiable for healthy individuals, further complicating the recruitment of an ideal control group. Finally, the study was focused on COPD; therefore, the performance of the approach in other lung and vascular diseases cannot be predicted. Further research on the applicability for other disease entities is warranted.

In conclusion, we demonstrated the feasibility of identifying COPD as a deviation from normality. The produced region anomaly scores provide visual representations of local deviations and can potentially serve as surrogate markers to disentangle COPD phenotypes and early identification of individuals at risk. The subject-wise anomaly score provides an interpretable metric that explains common clinical and radiological manifestations. Future work will focus on generalizability to other datasets and lung diseases, as well as longitudinal analysis.

### Supplementary Information

Below is the link to the electronic supplementary material.

Supplementary file1 (PDF 749 KB)

## Abbreviations

COPD: Chronic obstructive pulmonary disease
COPDGene: Genetic Epidemiology of COPD
COSYCONET: SYstemic consequences-COmorbidities NETwork
Exp: Expiratory CT images
ExpR: Registered expiratory CT images
FEV_1_: Forced expiratory volume in 1 s
FVC: Forced vital capacity
GOLD: Global Initiative for Chronic Obstructive Lung Disease
Insp: Inspiratory CT images
LAA-856: Percentage of lung voxels with CT attenuation less than − 856 HU on expiration
LAA-950: Percentage of lung voxels with CT attenuation less than − 950 HU on inspiration
SGRQ: St. George’s Respiratory Questionnaire

## Declarations

### Guarantor

The scientific guarantor of this publication is Prof. Dr. Klaus Maier-Hein.

### Statistics and biometry

One of the authors has significant statistical expertise: Vivienn Weru, Division of Biostatistics, German Cancer Research Center (DKFZ), Heidelberg, Germany.

### Study subjects or cohorts overlap

The used cohorts (COPDGene and COSYCONET) are open access, therefore have been reported in several previous studies, including our own:

Almeida, S.D. et al (2023). cOOpD: reformulating COPD classification on chest CT scans as anomaly detection using contrastive representations. In: Greenspan, H., et al Medical Image Computing and Computer Assisted Intervention – MICCAI 2023. MICCAI 2023. Lecture Notes in Computer Science, vol 14224. Springer, Cham. https://doi.org/10.1007/978-3-031-43904-9_4.

### Methodology

• retrospective

• observational

• multicenter study
