# Supplementary material for: Prediction of disease severity in COPD: a deep learning approach for anomaly-based quantitative assessment of chest CT
Source: Eur Radiol. 2023 Dec 27;34(7):4379–92. doi: 10.1007/s00330-023-10540-3 (PMC11213737; doi:10.1007/s00330-023-10540-3)
Supplement: Supplementary file 1 — Supplementary file1 (PDF 749 KB) [file 330_2023_10540_MOESM1_ESM.pdf]

Supplementary Materials

All experiments were implemented in PyTorch and Pytorch-Lightning, using a NVIDIA GeForce RTX 2080 Ti GPU.

S-1. Quantitative Chest Computed Tomography acquisition details

COSYCONET: Paired non-enhanced low-dose chest CT was acquired from several multi-detector row scanner (Somatom Definition AS 40/64/Flash 128, Siemens Healthineers, Erlangen, Germany; GE Lightspeed VCT 64/ GE Optima 64; Philips Brilliance 64/ iCT 256) in a standardized fashion in inspiratory and end-expiratory breath-hold with slice collimation 0.6-0.625 mm, pitch 0.6-1.0, tube voltage 120 kVp, tube current time product 30-35 mAs, and a total effective dose <3.5 mSv. Axial volumetric image reconstructions were performed using smooth convolution kernel at a slice thickness of 0.625-1.00 mm and a reconstruction interval of 0.5-0.75 mm. Dose modulation and iterative reconstruction were not applied. In-depth specifications are shown in Supplementary Table 1 and 2, taken from[1].

COPDGene: Paired non-enhanced chest CT scans were acquired using CT scanners from different manufacturers (GE LS 16, GE VCT-64, GE HD 750, Philips 16 Slice, Philips 40 Slice, Philips 64 Slice, Siemens Sensation-16, Siemens Sensation-64, Siemens Definition Dual Source 64, Siemens Definition AS++ 128, Siemens Definition Flash 128, Siemens Biograph 40, and Siemens Definition AS++ 128). The scanning protocol employed in COPDGene involved acquiring inspiratory and expiratory breath-hold CT scans. The specific acquisition parameters varied depending on the scanner model. However, common parameters included a rotation time of 0.5 seconds, detector configurations of 16×0.625, 64×0.625, or 128×0.625, pitch values ranging from 0.923 to 1.375, and speeds of 0.5 mm/rotation. The tube voltage was consistently set at 120 kVp across all scanners, while the tube current-time product varied between 50 and 200 mAs, depending on the specific scan and phase. Dose modulation techniques were not used. Regarding the image reconstructions, different algorithms and parameters were applied. The reconstructions were performed using various algorithms such as BONE, Detail (D), and B, B46f, with slice thicknesses of 0.625 to 0.9 mm and intervals of 0.45 to 0.5 mm. The field of view (DFOV) was generally tailored to lung imaging.
The above paragraph is a general description based on the information provided in the original COPDGene study publications[2]. The specific details and variations in the scanning protocols might require further analysis of the study documentation for complete accuracy.

S-2. Pre-processing

Spatial alignment of paired Insp and Exp images: Exp CT scans were registered to the correspondent Insp CT scan for each patient resulting in the registered Exp CT Scans (ExpR). Having the Insp image as the fixed image, an adaptation of [3] using *SimpleElastix* [4] was employed. *SimpleElastix* is an open-source deformable image registration library. Our adaptation employs a combination of an affine and a nonrigid B-spline transformation to model the spatial relationship between the two images, iteratively optimizing with a normalized correlation criterion with a bending energy penalty as the objective function. The optimized transformation matrix aligns the original Exp image to the Insp image space.

Lung parenchyma segmentation: Since both images per patient (Insp and ExpR) are on the same physical space, lung parenchyma and lobe masks only needed to be generated on the Insp physical space. It's important to note that the generated masks do not need to be a perfect segmentation, as these are only needed to extract 3D patches. Lung masks on the Insp image space were obtained by nnU-Net[5]. nnU-Net is a deep-learning based network designed for generic high performance biomedical image segmentation, reflecting a state-of-the-art segmentation methodology, well suited for a variety of tasks. It builds on a self-configuration framework for data pre-processing, network architecture, model training and segmentation postprocessing, thereby surpassing most traditional approaches for handcrafted supervised training of segmentation models. Training data segmentation masks were obtained from YACTA[6–8], an intensity based validated method on the entire COSYCONET dataset. Inference was then performed on the COPDGene dataset.

Intensity normalization: Based on[9], the inter-site scanner differences were accounted for by rescaling the intensities to a range from zero (air) to one (tissue). The air and tissue intensities were obtained by taking the median intensity of the trachea and the aorta, after binary eroding the masks, respectively. Trachea and aorta mean intensities were obtained by segmenting the correspondent areas on Insp scans. These were obtained using the nnU-Net pre-trained model *Task_055_SegTHOR*. Images were then resampled to isotropic resolution (0.5 mm).

Patch extraction: Finally, volumetric patches (50 cubic voxels) containing > 70% of the lung were extracted from the lung parenchyma of aligned Insp and ExpR CT images. The chosen size covered the secondary pulmonary lobule, the basic unit of lung structure[10].

S-3. Self-supervised contrastive learning: From patches to latent representations

Informative latent representations are obtained with SimCLR[11] as a framework for contrastive representations using a ResNet34 as encoder. This is based on the principle of contrasting samples against each other to learn semantic information. While similar semantic samples are attracted to each other, by minimizing a contrastive loss, dissimilar samples are moved away. As there are no patch-level labels available, the strategy of this self-supervised setting is augmenting a patch twice, using a set of optimal transformations. These were set based on the findings from Zhou, Z. et al.[12] on chest CT scans: non-linear transformations to model abnormal intensity appearance; texture transformations to learn untypical texture and boundaries by local pixel shuffling; and context as discontinuity by inner and outer cutouts. Training configuration for this pretext task was performed as described by Almeida et al.[13] and Lueth et al.[14]: based on the COPDGene training set, a maximum of 100 unlabeled 3D lung patches per subject were employed for 100 epochs, using the Adam Optimizer, learning rate of 1e-4, Cosine Annealing[15], 10 Warm-up Epochs and a weight decay of 1e-6, the temperature of the contrastive loss is 0.5[11]. Maximum 100 patches per subject was set based on previous experiments that showed that using all patches available per subject can introduce redundancy and increased computational costs.

S-4. Modeling the representative distribution model of normal appearing lung

Aiming to explore the potential of normal and unlabeled features, the pretrained contrastive model was employed to generate patch-level representations of purely normal patches. Patch normality was defined by %Emphysema<1% strictly applied to the "control" group (healthy never-smoker individuals and individuals with minimal disease, minimal emphysema and

without airflow limitation [GOLD 0]), a very restrictive bound to guarantee that no intensity alterations could be present in the definition of normality. %Emphysema was defined as the percentage of low attenuation areas less than a threshold of -950 Hounsfield units (%LAA-950). Then, a generative model is fitted on the feature space of these normal appearing patches, as to estimate the representation likelihood.
We employed a Gaussian Mixture Model (GMM) and Normalizing Flows (NF) as generative models, without any transformations. Experiments were done using GMM with 1, 2, 4 and 8 components (K). Further specifications can be found in [13] and[14].

S-5. Detecting COPD as anomalies, patient level prediction, visualization maps

During inference, each patch is given an anomaly score based on the negative log likelihood $s(x_i)=-log(p(f(x_i)))$. Patient-level scores are obtained by aggregating the patch-level scores. The following aggregation strategies were experimented: mean, median, third quartile (Q3), percentile 95 (p95), percentile 99 (p99) and maximum value (max). Final aggregation strategy was chosen based on the highest AUC on three runs on the validation set, for all the input configurations (Insp 0%, Insp 20%, InspExpR 0%, InspExpR 20%). Once the aggregation strategy per input configuration was chosen, it was applied three times on the internal and external test set.
Lung anomaly maps were constructed based on the negative log likelihood score, normalized by the min-max normalization corresponding to the 5th and 95th percentiles of the corresponding test set.

S-6. Compared supervised methods (voxel-based and representation-based)
We compared the performance of our proposed method to four supervised learning methods: three voxel-based (PatClass+RNN, MIL+RNN, MIL+Att) and one representation-based (ReContrastive).

<u>PatClass+RNN:</u> A supervised end-to-end Patch Classifier with a recurrent neural network (RNN) as aggregation to obtain patient-level scores. The model receives as input a 3D patch, having the same label as the global patient label (0 or 1). As output, the model produces a probability at the patch-level, of being diseased or not. This probability is then aggregated per patient. Several aggregation strategies were experimented, but RNN, as described by[16], showed to be the best performing one on the validation set. Here, the S=10% most suspicious patches were sequentially passed to the RNN to predict the final patient-level classification. As an encoder we used a 3D ResNet34, which was trained for 100 epochs, with a batch size of 64, using the Adam Optimizer, learning rate of 1e-4, Cosine Annealing[15], with cross entropy loss. As transformations, elastic, rotation, scaling, random cropping, mirroring, gaussian noise and gaussian blur were employed with a probability of 5%.

<u>MIL+RNN:</u> A supervised multiple instance learning (MIL) strategy with a recurrent neural network as aggregation, as proposed by[16]. Given a bag of instances (where the bag is a patient and the instances are 3D patches), all instances are classified and ranked according to their probability of being positive (diseased). If the bag is positive (which means that the patient-level label is diseased), the probability of being positive of the highest-ranked instance should be very close to 1; if the bag is negative (normal), the highest ranked instance should have a very low probability, approaching 0. Therefore, the task here is to learn the optimal instance (patch) level representation that can linearly discriminate patches that contribute to a diseased

global label, from those patches that do not. The implementation details are as described in the original paper, with the exception of the encoder (3D ResNet34). S (probability) was set to 10%.

MIL+Att: A supervised MIL with an attention layer. Similar to the MIL+RNN method, this MIL builds on the same strategy but employs a latter layer with an attention mechanism. This layer provides a measure of the contribution of each patch to the overall patient label. The overall idea is identical to Sun et al. COPD detection model[17], except we build on the 3D patch notion, while they selected slices from the original CT. Overall configuration details were as described by[18], with the adaptations to 3D, and using a 3D ResNet34 as encoder.

ReContrastive: A supervised CNN classifier operating on the latent representations produced by our proposed contrastive DL model (cOOpD). Patch-level representations of the two classes (“control” and “diseased”) are generated by the pretrained model in S-3. Considering that the anatomical context was lost in the creation of these individual unconnected patches, we handle this trait by reconstructing the representation vectors to their original location in the lung. This produces a 4D image at the patient-level, where the 4th dimension is the length of the representation vector. Next, we apply a LeNet[19] as our supervised encoder which outputs the two possible classes. The assumption is that this image composed of "anatomically-composed" representations is highly informative, therefore a simple encoder should be able to capture the critical features for the classification. This was performed for 500 epochs using the Stochastic Gradient Descent Optimizer, a learning rate of 1e-2, Cosine Annealing and a weight decay of 3e-5. As transformations on the reconstructed latent representation image, we employed a combination of random cropping, random scaling, random mirroring, rotations, and Gaussian blurring.

S-7. Model performance

Model performance for COPD binary classification was assessed using Area Under Receiver Operator Curve (AUC) and Area Under Precision Recall Curve (AUPRC) as the default multi-threshold metric for classification. AUC is used as the main evaluation metric since it is less sensitive to class balance changes. Final method configurations were tuned on the evaluation set, based on the highest AUC on three experiment runs. Supplementary Table 3 further shows all methods’ performance on the internal and external test sets, for all inputs.

Supplementary references

1. Kellerer, C. *et al.* Prediction of lung emphysema in COPD by spirometry and clinical symptoms: results from COSYCONET. *Respir Res* **22**, 242 (2021).
2. Regan, E. A. *et al.* Genetic Epidemiology of COPD (COPDGene) Study Design. *COPD: Journal of Chronic Obstructive Pulmonary Disease* **7**, 32–43 (2011).
3. Staring, M., Klein, S., Reiber, J. H. C., Niessen, W. & Stoel, B. Pulmonary image registration with elastix using a standard intensity-based algorithm. in 73–79 (2010).
4. Marstal, K., Berendsen, F., Staring, M. & Klein, S. SimpleElastix: A User-Friendly, Multi-lingual Library for Medical Image Registration. in *2016 IEEE Conference on Computer Vision and Pattern Recognition Workshops (CVPRW)* 574–582 (IEEE, 2016). doi:10.1109/CVPRW.2016.78.
5. Isensee, F., Jaeger, P. F., Kohl, S. A. A., Petersen, J. & Maier-Hein, K. H. nnU-Net: a self-configuring method for deep learning-based biomedical image segmentation. *Nat Methods* **18**, 203–211 (2021).
6. Kahnert, K. *et al.* Standardized airway wall thickness Pi10 from routine CT scans of COPD patients as imaging biomarker for disease severity, lung function decline, and mortality. *Ther Adv Respir Dis* **17**, 175346662211486 (2023).
7. Konietzke, P. *et al.* Validation of automated lobe segmentation on paired inspiratory-expiratory chest CT in 8-14 year-old children with cystic fibrosis. *PLoS ONE* **13**, e0194557 (2018).
8. Konietzke, P. *et al.* Quantitative CT detects progression in COPD patients with severe emphysema in a 3-month interval. *Eur Radiol* **30**, 2502–2512 (2020).
9. Kim, S. S. *et al.* Improved correlation between CT emphysema quantification and pulmonary function test by density correction of volumetric CT data based on air and aortic density. *European Journal of Radiology* **83**, 57–63 (2014).
10. Webb, W. R. Thin-Section CT of the Secondary Pulmonary Lobule: Anatomy and the Image—The 2004 Fleischner Lecture. *Radiology* **239**, 322–338 (2006).
11. Chen, T., Kornblith, S., Norouzi, M. & Hinton, G. A Simple Framework for Contrastive Learning of Visual Representations. (2020) doi:10.48550/ARXIV.2002.05709.
12. Zhou, Z. *et al.* Models Genesis: Generic Autodidactic Models for 3D Medical Image Analysis. in *Medical Image Computing and Computer Assisted Intervention – MICCAI 2019* (eds. Shen, D. et al.) vol. 11767 384–393 (Springer International Publishing, 2019).
13. Almeida, S. D. *et al.* cOOpD: Reformulating COPD Classification on Chest CT Scans as Anomaly Detection Using Contrastive Representations. in *Medical Image Computing and Computer Assisted Intervention – MICCAI 2023* (eds. Greenspan, H. et al.) vol. 14224 33–43 (Springer Nature Switzerland, 2023).
14. Lüth, C. T. *et al.* CRADL: Contrastive Representations for Unsupervised Anomaly Detection and Localization. (2023) doi:10.48550/ARXIV.2301.02126.
15. Loshchilov, I. & Hutter, F. SGDR: Stochastic Gradient Descent with Warm Restarts. (2016) doi:10.48550/ARXIV.1608.03983.
16. Campanella, G. *et al.* Clinical-grade computational pathology using weakly supervised deep learning on whole slide images. *Nat Med* **25**, 1301–1309 (2019).
17. Sun, J. *et al.* Detection and staging of chronic obstructive pulmonary disease using a computed tomography–based weakly supervised deep learning approach. *Eur Radiol* **32**, 5319–5329 (2022).
18. Ilse, M., Tomczak, J. M. & Welling, M. Attention-based Deep Multiple Instance

Learning. (2018) doi:10.48550/ARXIV.1802.04712.
19. Lecun, Y., Bottou, L., Bengio, Y. & Haffner, P. Gradient-based learning applied to document recognition. *Proc. IEEE* **86**, 2278–2324 (1998).

Supplementary Figure Captions

Supplementary Figure 1: Paired inspiratory and expiratory CT images were acquired per subject. Pre-processing comprises image registration, image segmentation, image normalization and patch-extraction. Expiratory images are aligned to inspiratory ones, so that both are in the same physical space. Lung, trachea and aorta are segmented using masks from YACTA to train nnU-Net models (lung) and using nnU-Net pretrained model for the trachea and aorta. Intensity normalization and resampling was applied. Volumetric patches (50 cubic voxels) were extracted from the lung parenchyma under four configurations: Insp 0% (overlapping patches), Insp 20%, InspExpR 0% and InspExpR 20%.

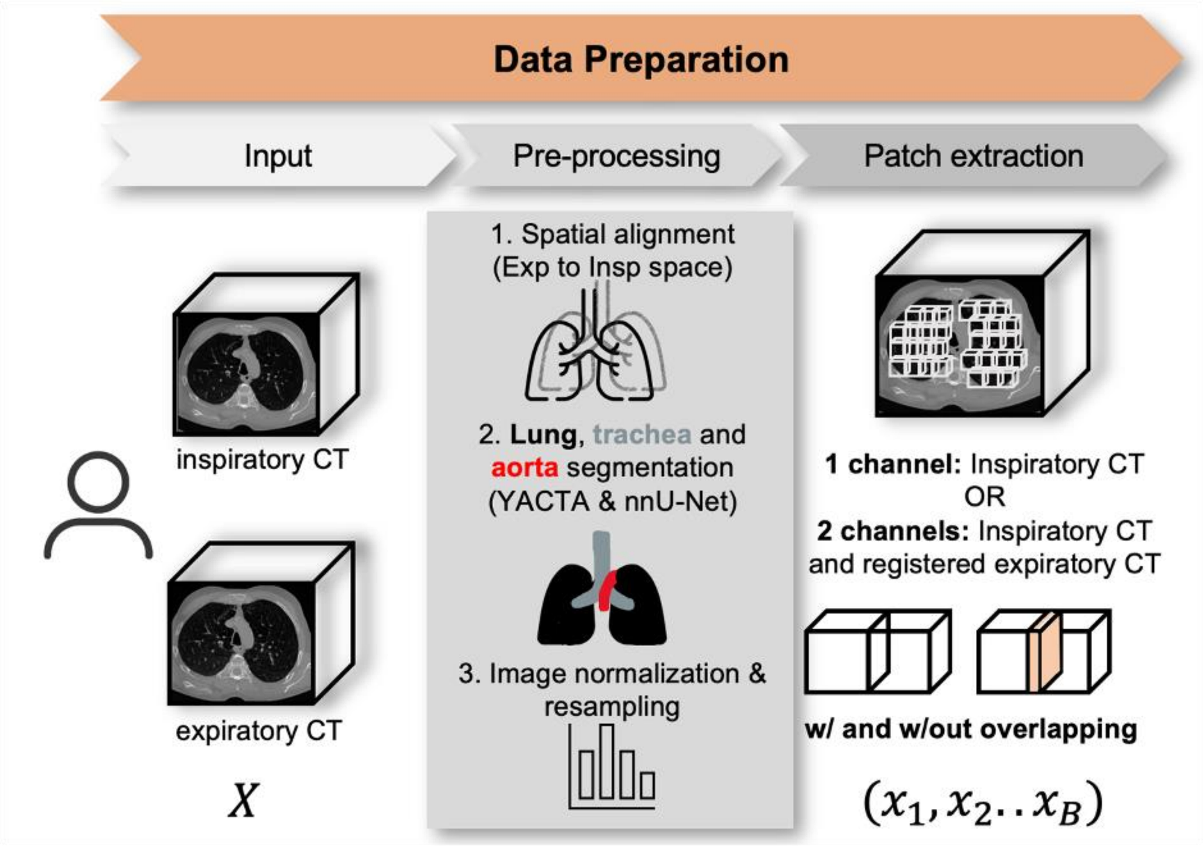

Supplementary Figure 2: COSYCONET forest-plots of standardized beta values of the linear mixed effects models to predict the St. George's Respiratory Questionnaire (A), 6-minutes walking test (B), FEV_1 (C), FEV_1/FVC (D), %Emphysema (E) and %Air Trapping (F). Baseline models (without the anomaly score) are colored in gray, while baseline + anomaly score models are colored in black. (p<0.05*/0.01**/0.001***).
BMI = Body Mass Index; FEV_1 = Forced Expiratory Volume in 1 second; FEV_1/FVC = FEV_1-to-forced vital capacity ratio.

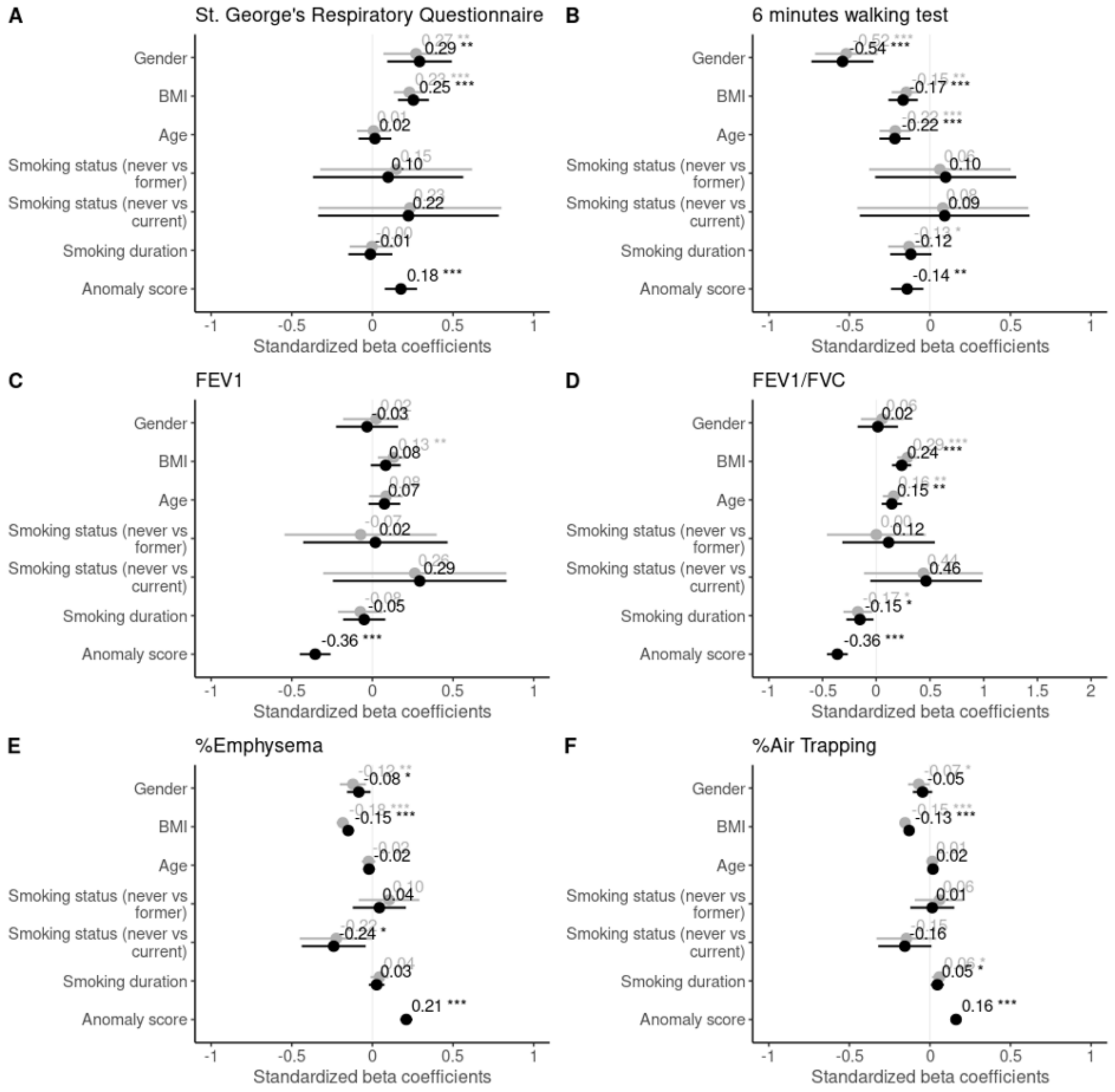

Supplementary Figure 3: Distribution of patient-wise anomaly scores from COSYCONET versus %Emphysema (A) and %Air Trapping (B), with reported Pearson's correlation coefficient (r), colored by GOLD stage. Subjects who experienced severe exacerbations in the past year were significantly different than those who did not experience in terms of the anomaly score (p<0.01, Wilcoxon test) (C). The distribution of the anomaly score did not differ among the mMRC dyspnea classes (p=0.1144, Jonckheere-Terpstra) (D).

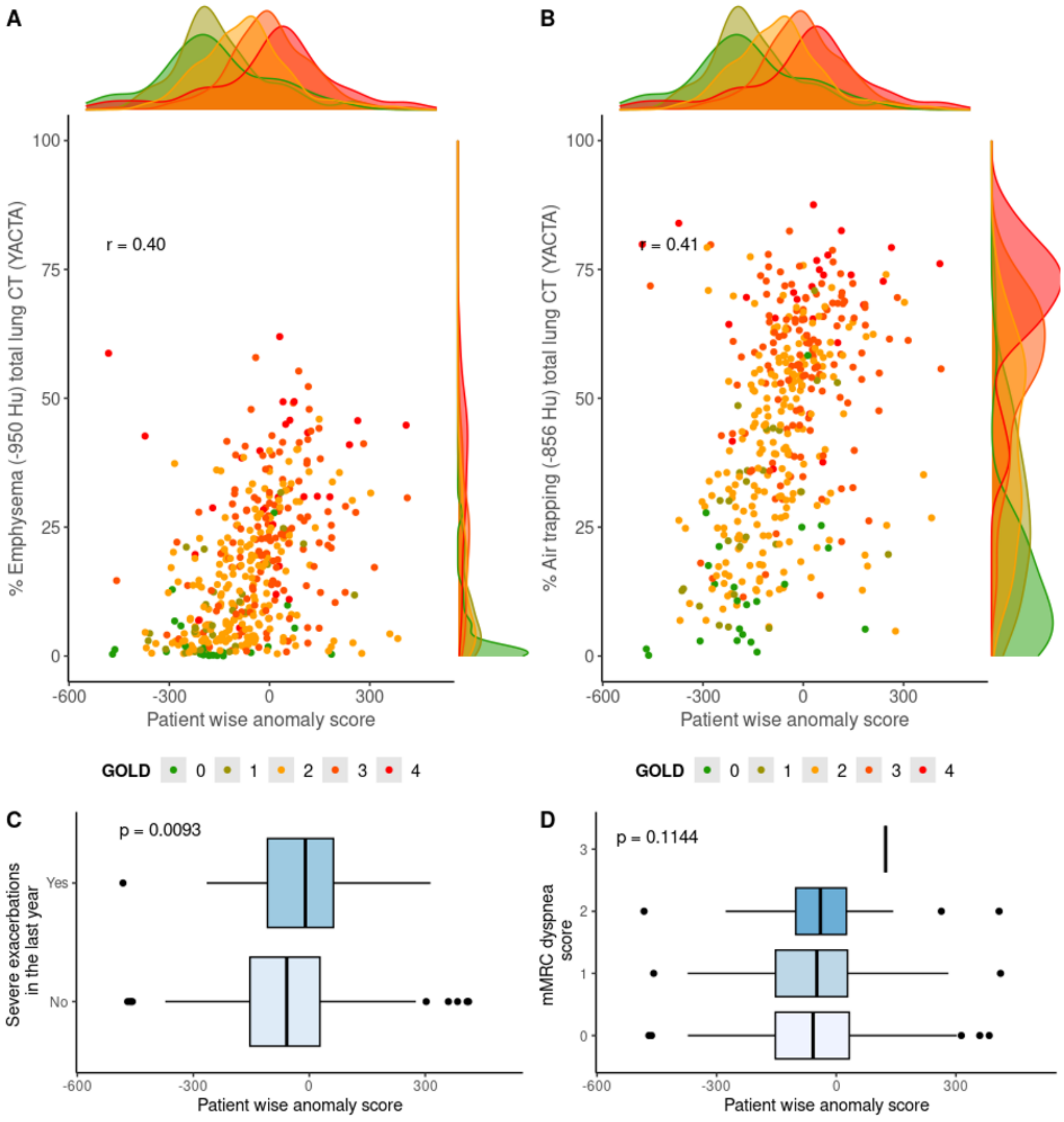

Supplementary Tables Captions

Supplementary Table 1: COSYCONET study protocol.

Acquisition	Orientation	FOV	Slice thickness (mm)	Interval (mm)	Convolution kernel*
**Inspiratory**	axial	lung	1.25-1.50	0.70-0.75	B70f/LUNG/L
**Inspiratory**	axial	lung	0.625-1.00	0.50	B30f/SOFT/B
**Inspiratory**	axial	including soft tissue of torso	0.625-1.00	0.50	B30f/SOFT/B
**Expiratory**	axial	lung	0.625-1.00	0.50	B30f/SOFT/B

Supplementary Table 2: Details on the scanner models for COSYCONET. Vendor-specific generic names for Siemens/GE/Philips.

**Scanner models**	Siemens Definition AS 40/ 64/ Flash 128 GE Lightspeed VCT 64/ GE Optima 64/ Philips Brilliance 64/ iCT 256
Scan Type	Spiral
Rotation Time (s)	0.33 – 0.50 s
Collimation	40 / 64 / 128 x 0.6-0.625 mm
Pitch	0.6-1.0
kVp	120 kVp
mA	30 - 35 eff. mAs
Dose modulation	Off
Matrix	512 x 512
Calibration phantom	Air / water phantom / CatPhan
Max. eff dose/scan	< 1.75 mSv
Max. eff. overall dose	< 3.50 mSv

Supplementary Table 3: Classification performance on the internal and external test sets for all inputs and for all compared methods. Note - Mean ± standard deviation of the Area Under the Receiver Operating Curve (AUC) and Area Under the Precision Recall Curve (AUPRC) in % of 3 runs on the internal (COPDGene) and external (COSYCONET) test sets. Compared methods are the anomaly detection method (cOOpD), Performance metric (AUC) for the internal (COPDGene) and external (COSYCONET) test sets, for four different input configurations, for the anomaly detection method (cOOpD) and four supervised deep-learning methods: end-to-end Patch Classifier with a recurrent neural network [PatClass+RNN], a multiple instance learning [MIL] with RNN as aggregation [MIL+RNN], an attention based MIL [MIL+Att]) and one representation-based [ReContrastive]. The best performing generative model for cOOpD is shown: Normalizing Flow (NF), Gaussian Mixture Model with two or four components (GMM2, GMM4). The best performing aggregation strategy is shown: mean, third quartile (Q3). Best results are highlighted in bold.

Input	Method	COPDGene		COSYCONET	
		AUC	AUPRC	AUC	AUPRC
Insp 0%	PatClass + RNN	0.63 ± 0.05	0.78 ± 0.01	0.64 ± 0.05	0.96 ± 0.00
	MIL + Att	0.54 ± 0.02	0.73 ± 0.02	0.54 ± 0.05	0.94 ± 0.01
	MIL + RNN	0.59 ± 0.03	0.76 ± 0.01	0.64 ± 0.05	0.96 ± 0.01
	ReContrastive	0.78 ± 0.00	0.88 ± 0.00	0.74 ± 0.01	**0.97 ± 0.00**
	cOOpD (NF Q3)	**0.83 ± 0.00**	**0.89 ± 0.00**	**0.76 ± 0.00**	0.96 ± 0.00
Insp 20%	PatClass + RNN	0.68 ± 0.01	0.81 ± 0.01	0.72 ± 0.02	0.97 ± 0.00
	MIL + Att	0.50 ± 0.02	0.72 ± 0.01	0.52 ± 0.06	0.93 ± 0.01
	MIL + RNN	0.63 ± 0.02	0.78 ± 0.01	0.68 ± 0.08	0.96 ± 0.01
	ReContrastive	0.78 ± 0.00	0.88 ± 0.00	0.68 ± 0.02	0.96 ± 0.00
	cOOpD (NF median)	**0.84 ± 0.00**	**0.90 ± 0.00**	**0.76 ± 0.01**	**0.98 ± 0.00**
InspExpR 0%	PatClass + RNN	0.77 ± 0.01	0.86 ± 0.01	0.56 ± 0.01	0.82 ± 0.22
	MIL + Att	0.60 ± 0.03	0.77 ± 0.02	0.55 ± 0.01	0.94 ± 0.01
	MIL + RNN	0.75 ± 0.01	0.84 ± 0.00	0.64 ± 0.03	**0.96 ± 0.00**
	ReContrastive	0.79 ± 0.00	**0.87 ± 0.00**	0.53 ± 0.01	0.95 ± 0.00
	cOOpD (GMM2 mean)	**0.84 ± 0.01**	**0.87 ± 0.02**	**0.73 ± 0.01**	0.95 ± 0.00
InspExpR 20%	PatClass + RNN	0.76 ± 0.00	0.86 ± 0.00	0.56 ± 0.01	0.95 ± 0.00
	MIL + Att	0.66 ± 0.03	0.81 ± 0.02	0.58 ± 0.01	0.95 ± 0.01

	MIL + RNN	0.73 ± 0.01	0.85 ± 0.00	0.60 ± 0.04	0.96 ± 0.00
	ReContrastive	0.80 ± 0.00	0.89 ± 0.00	0.53 ± 0.01	0.95 ± 0.00
	cOOpD (GMM4 mean)	**0.84 ± 0.00**	**0.90 ± 0.00**	**0.68 ± 0.01**	**0.95 ± 0.00**

Supplementary Table 4: Decomposition of the Explained Variation in the Linear Mixed Effects Model for COPDGene**.** Note - Percentages of explained variance (R^2) for the individual predictors to the corresponding linear mixed effects models. Confidence intervals (CI) are also reported (95%). * %Emphysema and %Air Trapping were skewed, so a log-transformation was applied. R package: *explainedVariance* with 500 simulations.
BMI = Body Mass Index; SGRQ = St. George 's Respiratory Questionnaire; 6MWT = 6-minutes-walking-test; FEV_1 = Forced Expiratory Volume in 1 second; FEV_1/FVC = FEV_1-to-forced vital capacity ratio.

**Proportion of explained variation by fixed effects**				
**Dependent variable**	**individual explained variance**	**R^2 (%)**	**CI 2.5 (%)**	**CI 97.5 (%)**
SGRQ	gender	0,01	-0,07	0,56
	BMI	0,09	-0,18	0,84
	age	-0,49	-0,71	-0,10
	smoking status	1,05	0,06	2,76
	smoking duration	3,41	1,44	5,89
	**anomaly score**	**19,28**	**15,78**	**24,68**
	total	23,34	20,24	29,34
6MWT	gender	1,57	0,57	2,77
	BMI	2,53	1,23	4,21
	age	4,11	2,14	6,31
	smoking status	-0,62	-0,74	-0,14
	smoking duration	4,49	2,26	6,64
	**anomaly score**	**11,62**	**8,10**	**14,63**
	total	23,70	17,66	28,23
FEV_1	gender	-0,05	-0,06	0,19
	BMI	-0,15	-0,18	0,17
	age	-0,36	-1,45	0,85
	smoking status	0,11	-0,50	1,12
	smoking duration	5,80	3,42	8,50
	**anomaly score**	**38,51**	**34,81**	**43,68**

	total	43,86	40,51	49,06
FEV$_1$/FVC	gender	0,22	-0,04	0,82
	BMI	1,69	0,79	3,02
	age	2,15	0,66	4,07
	smoking status	0,14	0,47	1,19
	smoking duration	5,55	3,26	8,04
	**anomaly score**	**41,22**	**37,90**	**45,37**
	total	50,97	48,19	55,36
% Emphysema*	gender	4,16	2,70	5,95
	BMI	3,33	1,93	4,69
	age	0,71	-0,73	2,45
	smoking status	5,41	3,27	7,45
	smoking duration	1,61	0,32	3,03
	**anomaly score**	**27,77**	**22,77**	**31,62**
	total	42,98	36,51	47,33
% Air Trapping*	gender	1,84	0,97	2,96
	BMI	2,85	1,81	4,43
	age	6,76	4,56	9,70
	smoking status	2,63	1,43	4,22
	smoking duration	0,71	-0,56	2,35
	**anomaly score**	**40,19**	**36,65**	**44,50**
	total	54,98	52,23	59,39

Supplementary Table 5. Linear mixed effects model to predict several clinical (SGRQ, 6MWT, FEV$_1$, FEV$_1$/FVC) and radiological (%Emphysema, %Air Trapping) dependent variables using the produced anomaly score as a predictor, for the COSYCONET test cohort (n=446). Note - Models are adjusted for age, gender, BMI, smoking status, smoking duration and a random term for the study site. This model was compared with a baseline model that omits the anomaly score. Conditional R^2 is adjusted for the number of regressors added. p-values are reported per model and for the comparison between them and are corrected for multiple comparisons.
R^2 = overall conditional coefficient of determination; BMI = Body Mass Index; SGRQ = St. George 's Respiratory Questionnaire; 6MWT = 6-minutes-walking-test; FEV$_1$ = Forced Expiratory Volume in 1 second; FEV$_1$/FVC = FEV$_1$-to-forced vital capacity ratio.

dependent variable	predictor	adjusted conditional R^2	p-value	
SGRQ	age, gender, BMI, smoking status, smoking duration, (center)	0.08	p > .001	p > .001
	age, gender, BMI, smoking status, smoking duration, anomaly score, (center)	**0.11**	p < .001	
6MWT	age, gender, BMI, smoking status, smoking duration, (center)	0.24	p < .001	p > .001
	age, gender, BMI, smoking status, smoking duration, anomaly score, (center)	**0.24**	p < .001	
FEV$_1$	age, gender, BMI, smoking status, smoking duration, (center)	0.07	p > .001	p < .001
	age, gender, BMI, smoking status, smoking duration, anomaly score, (center)	**0.17**	p < .001	
FEV$_1$/FVC	age, gender, BMI, smoking status, smoking duration, (center)	0.13	p < .001	p < .001
	age, gender, BMI, smoking status, smoking duration, anomaly score, (center)	**0.27**	p < .001	
Emphysema %	age, gender, BMI, smoking status, smoking duration, (center)	0.22	p < .001	p < .001
	age, gender, BMI, smoking status, smoking duration, anomaly score, (center)	**0.43**	p < .001	
Air Trapping %	age, gender, BMI, smoking status, smoking duration, (center)	0.22	p < .001	p < .001
	age, gender, BMI, smoking status, smoking duration, anomaly score, (center)	**0.41**	p < .001	

Supplementary Table 6: Decomposition of the Explained Variation in the Linear Mixed Effects Model for COSYCONET. Note - Percentages of explained variance (R^2) for the individual predictors to the corresponding linear mixed effects models. Confidence intervals (CI) are also reported (95%) * %Emphysema and %Air Trapping were skewed, so a log-transformation was applied. R package: *explainedVariance* with 500 simulations.
BMI = Body Mass Index; SGRQ = St. George 's Respiratory Questionnaire; 6MWT = 6-minutes-walking-test; FEV_1 = Forced Expiratory Volume in 1 second; FEV_1/FVC = FEV_1-to-forced vital capacity ratio.

Proportion of explained variation by fixed effects				
**Dependent variable**	**individual explained variance**	**R^2 (%)**	**CI 2.5 (%)**	**CI 97.5 (%)**
SGRQ	gender	1,33	-0,06	4,42
	BMI	5,11	1,80	9,94
	age	-0,27	-0,35	0,91
	smoking status	-0,24	-0,52	2,21
	smoking duration	-0,27	-0,63	1,11
	anomaly score	**2,22**	**0,16**	**5,95**
	total	7,89	4,21	15,04
6MWT	gender	4,88	1,37	8,78
	BMI	1,91	0,20	4,94
	age	3,57	0,85	7,37
	smoking status	-0,51	-0,73	1,11
	smoking duration	1,45	-0,20	4,42
	anomaly score	**1,21**	**-0,17**	**4,01**
	total	12,50	8,09	19,87
FEV1post	gender	-0,18	-0,21	0,97
	BMI	0,67	-0,21	2,88
	age	0,31	-0,21	2,35
	smoking status	0,88	-0,30	4,22
	smoking duration	-0,27	-0,61	0,98

	anomaly score	**13,18**	**7,21**	**19,48**
	total	14,60	9,95	22,66
FEV/FVC	gender	-0,21	-0,23	0,86
	BMI	5,70	2,28	10,37
	age	1,46	0,00	4,15
	smoking status	0,51	-0,82	3,19
	smoking duration	0,43	-0,46	2,78
	anomaly score	**14,35**	**7,99**	**20,34**
	total	22,23	15,70	30,15
% Emphysema *	gender	1,09	-0,06	2,99
	BMI	9,14	4,84	13,19
	age	-0,13	-0,28	0,86
	smoking status	5,76	2,58	9,62
	smoking duration	-0,25	-0,67	0,78
	anomaly score	**21,19**	**12,94**	**26,96**
	total	36,80	27,39	42,93
% Air Trapping *	gender	0,00	-0,19	1,02
	BMI	9,53	4,97	13,84
	age	1,46	-0,02	3,74
	smoking status	2,69	0,44	6,13
	smoking duration	0,88	-0,15	3,28
	anomaly score	**20,70**	**14,24**	**27,11**
	total	35,26	28,18	42,74
